# SHEA position statement on pandemic preparedness for policymakers: introduction and overview

**DOI:** 10.1017/ice.2024.66

**Published:** 2024-07

**Authors:** Vincent P. Hsu, Sarah Haessler, David B. Banach, Lynne Jones Batshon, Westyn Branch-Elliman, Ghinwa Dumyati, Robin L. P. Jump, Anurag N. Malani, Trini A. Mathew, Rekha K. Murthy, Steven A. Pergam, Erica S. Shenoy, David J. Weber

**Affiliations:** 1 AdventHealth, Altamonte Springs, FL, USA; 2 Loma Linda University School of Medicine, Loma Linda, CA, USA; 3 Baystate Medical Center, Springfield, MA, USA; 4 University of Massachusetts Chan Medical School – Baystate, Springfield, MA, USA; 5 University of Connecticut School of Medicine, Farmington, CT, USA; 6 Yale School of Public Health, New Haven, CT, USA; 7 Society for Healthcare Epidemiology of America (SHEA), Arlington, VA, USA; 8 Veterans Affairs Boston Healthcare System, Boston, MA, USA; 9 Harvard Medical School, Boston, MA, USA; 10 University of Rochester Medical Center, Rochester, NY, USA; 11 Center for Community Health, Rochester, NY, USA; 12 Geriatric Research Education and Clinical Center (GRECC) at the Veterans Affairs Pittsburgh Healthcare System, Pittsburgh, PA, USA; 13 University of Pittsburgh School of Medicine, Pittsburgh, PA, USA; 14 Trinity Health Michigan, Ann Arbor, MI, USA; 15 HealthTAMCycle3, PLLC, Troy, MI, USA; 16 Corewell Health, Taylor, Michigan, USA; 17 School of Medicine, Wayne State University, Detroit, MI, USA; 18 Oakland University William Beaumont, Rochester, MI, USA; 19 Cedars-Sinai, Los Angeles, CA, USA; 20 David Geffen School of Medicine at UCLA, Los Angeles, CA, USA; 21 Fred Hutchinson Cancer Research Center, Seattle, WA, USA; 22 University of Washington, Seattle, WA, USA; 23 Seattle Cancer Care Alliance, Seattle, Washington, USA; 24 Massachusetts General Hospital, Boston, MA, USA; 25 Mass General Brigham, Boston, MA, USA; 26 University of North Carolina, Chapel Hill, NC, USA

## Abstract

Throughout history, pandemics and their aftereffects have spurred society to make substantial improvements in healthcare. After the Black Death in 14^th^ century Europe, changes were made to elevate standards of care and nutrition that resulted in improved life expectancy.^1^ The 1918 influenza pandemic spurred a movement that emphasized public health surveillance and detection of future outbreaks and eventually led to the creation of the World Health Organization Global Influenza Surveillance Network.^2^ In the present, the COVID-19 pandemic exposed many of the pre-existing problems within the US healthcare system, which included (1) a lack of capacity to manage a large influx of contagious patients while simultaneously maintaining routine and emergency care to non-COVID patients; (2) a “just in time” supply network that led to shortages and competition among hospitals, nursing homes, and other care sites for essential supplies; and (3) longstanding inequities in the distribution of healthcare and the healthcare workforce. The decades-long shift from domestic manufacturing to a reliance on global supply chains has compounded ongoing gaps in preparedness for supplies such as personal protective equipment and ventilators. Inequities in racial and socioeconomic outcomes highlighted during the pandemic have accelerated the call to focus on diversity, equity, and inclusion (DEI) within our communities. The pandemic accelerated cooperation between government entities and the healthcare system, resulting in swift implementation of mitigation measures, new therapies and vaccinations at unprecedented speeds, despite our fragmented healthcare delivery system and political divisions. Still, widespread misinformation or disinformation and political divisions contributed to eroded trust in the public health system and prevented an even uptake of mitigation measures, vaccines and therapeutics, impeding our ability to contain the spread of the virus in this country.^3^ Ultimately, the lessons of COVID-19 illustrate the need to better prepare for the next pandemic. Rising microbial resistance, emerging and re-emerging pathogens, increased globalization, an aging population, and climate change are all factors that increase the likelihood of another pandemic.^4^

## Background

Throughout history, pandemics and their aftereffects have spurred society to make substantial improvements in healthcare. After the Black Death in 14^th^ century Europe, changes were made to elevate standards of care and nutrition that resulted in improved life expectancy.^1^ The 1918 influenza pandemic spurred a movement that emphasized public health surveillance and detection of future outbreaks and eventually led to the creation of the World Health Organization Global Influenza Surveillance Network.^2^ In the present, the COVID-19 pandemic exposed many of the pre-existing problems within the US healthcare system, which included (1) a lack of capacity to manage a large influx of contagious patients while simultaneously maintaining routine and emergency care to non-COVID patients; (2) a “just in time” supply network that led to shortages and competition among hospitals, nursing homes, and other care sites for essential supplies; and (3) longstanding inequities in the distribution of healthcare and the healthcare workforce. The decades-long shift from domestic manufacturing to a reliance on global supply chains has compounded ongoing gaps in preparedness for supplies such as personal protective equipment and ventilators. Inequities in racial and socioeconomic outcomes highlighted during the pandemic have accelerated the call to focus on diversity, equity, and inclusion (DEI) within our communities. The pandemic accelerated cooperation between government entities and the healthcare system, resulting in swift implementation of mitigation measures, new therapies and vaccinations at unprecedented speeds, despite our fragmented healthcare delivery system and political divisions. Still, widespread misinformation or disinformation and political divisions contributed to eroded trust in the public health system and prevented an even uptake of mitigation measures, vaccines and therapeutics, impeding our ability to contain the spread of the virus in this country.^3^ Ultimately, the lessons of COVID-19 illustrate the need to better prepare for the next pandemic. Rising microbial resistance, emerging and re-emerging pathogens, increased globalization, an aging population, and climate change are all factors that increase the likelihood of another pandemic.^4^

## Rationale for a pandemic preparedness policy workgroup from an epidemiologic perspective

Within the healthcare sector, the COVID-19 pandemic had an outsized effect on healthcare professionals within the field of infection prevention and epidemiology. Epidemiologists and infection preventionists are leaders that use science to guide strategies in reducing the spread of transmissible disease in healthcare and public health settings. The Society for Healthcare Epidemiology of America (SHEA) aims to improve public health and reduce healthcare-associated infections by establishing evidence-based infection prevention practices in healthcare settings. Historically, SHEA has accomplished its aims by providing clinical guidance documents and expert consensus statements, but recently has increased participation in advocacy efforts designed for local, state, and federal policymakers. During the pandemic, SHEA spearheaded and issued a Multi-society Statement on COVID-19 Vaccination as a Condition of Employment,^5^ published toolkit guidance for management of COVID-19 and vaccination in various healthcare settings, and convened webinars, town halls, and podcasts to further empower healthcare epidemiologists and infection preventionists to mitigate the effects of the pandemic on their institutions and communities.^6^

In late 2021, a SHEA workgroup was convened at the request of the SHEA Public Policy and Government Affairs Committee to specifically address ongoing needs related to pandemic preparedness by writing a series of position papers that also serve as a template for advocacy efforts. This workgroup consists of SHEA members throughout the country with expertise in acute care, long-term care, and ambulatory care, in both academic and community settings and specifically focused on healthcare systems from an epidemiologic perspective.

**Figure 1.**
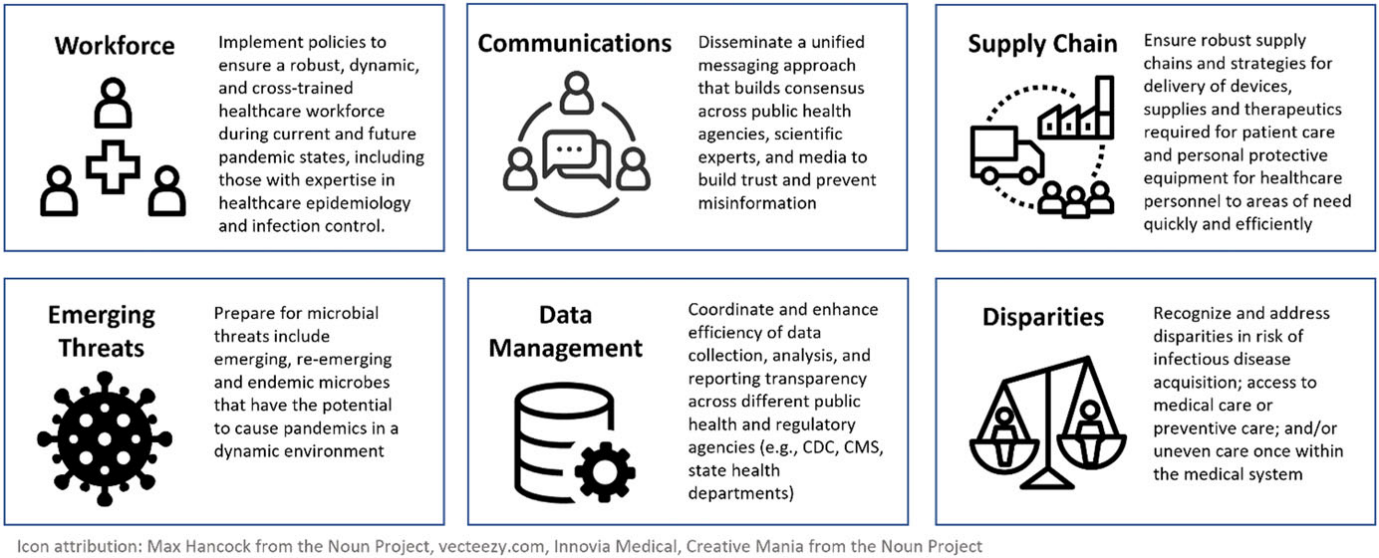
Topics selected by the SHEA Pandemic Preparedness workgroup for policymakers.

Using a combination of literature searches and expert opinion derived from shared experiences during the COVID-19 pandemic, the workgroup developed specific topics for these position statements along with a series of recommendations for policymakers (Figure 1):

1. Healthcare workforce shortage. An understaffed healthcare workforce was further exposed during times of increased healthcare utilization, such as during the Delta and Omicron waves. Cross training additional professionals in infection prevention and epidemiology, as well as strengthening the relationships between public health, frontline workforce, and infection prevention specialists will be necessary to ensure appropriate responses during similar crises.
2. Communication. Healthcare epidemiologists and infection preventionists need to be supported as effective communicators, ensuring bidirectional and coordinated flow of information to and from governments, public health agencies, healthcare institutions, providers and patient. Formal training and standardized communication can assist healthcare epidemiologists, infection preventionists, and public health professionals to adapt to the rapid evolution of an outbreak and stem the flow of misinformation and disinformation during public health emergencies.
3. Preparedness in the medical supply chain. While maintaining an uninterrupted supply of medical equipment involves preparedness on federal, state, and institutional levels, it also relies upon a unique and ever-changing global supply chain, which has a direct impact on the ability to procure a reliable supply of personal protective equipment at the institutional level during times of disruption. Adequate and timely access to medical supplies for patient care and personal protective equipment for healthcare personnel requires a transparent and accessible national stockpile.
4. Preparedness for emerging infectious threats. An improved network of surveillance systems and multiple health agencies must be coordinated to detect potential pandemics at an early stage. Additional research for pandemic preparedness, such as in vaccine development and understanding the interplay between animals, humans, and the environment remains crucial. Healthcare must play a role not only in training providers to respond to these threats, but in reducing the emergence of such pathogens through antimicrobial stewardship, climate change mitigation, and reducing socioeconomic disparities.
5. Healthcare data. Streamlining and reporting of data between healthcare entities and multiple agencies must be thoughtfully redesigned and implemented to ensure that the greatest amount of standardized data are collected and organized. This also means data are as widely available and transparent as possible, while able to remain relevant and funded for the long term, regardless of the next pandemic pathogen.
6. Health disparities. Addressing inequities in COVID-19 outcomes, vaccine uptake, and provision of therapeutic agents among persons of color, marginalized populations, and vulnerable groups such as those who reside in skilled nursing facilities or rural areas is paramount to reducing disparities in healthcare outcomes. Healthcare epidemiologists and professionals in infection prevention have a duty to address the need for equitable outcomes, not only in times of crisis, but during routine surveillance and prevention for healthcare-associated infections. This topic will be released as a document separate from this series through the SHEA DEI committee.

The workgroup recognizes that implementing these recommendations in their entirety requires major changes within the healthcare system. Nonetheless, any movement toward adopting recommended policy actions provides an additional step ahead toward future pandemic preparedness. Another pandemic is certain to threaten the world and we must learn from this one, using our experiences in epidemiology and the scientific data to guide and prepare our policymakers.
